# The effects of disease-modifying therapies in SMA-5q type 1 and the importance of early diagnosis of the disease

**DOI:** 10.1055/s-0044-1800927

**Published:** 2024-12-15

**Authors:** Edmar Zanoteli

**Affiliations:** 1 Universidade de São Paulo, Faculdade de Medicina, Departamento de Neurologia, São Paulo SP, Brazil.

Spinal muscular atrophy linked to chromosome 5 (SMA-5q) is an autosomal recessive neurodegenerative disorder caused by mutations in the *SMN1* gene. 1 It leads to different grades of muscle hypotonia, proximal weakness, and bulbar and respiratory insufficiency. Based on their maximum motor ability and age of onset, patients are classified into five groups, from zero to four. 2 Type 1 SMA-5q is the most common form and is characterized by an early onset of the disease (0–6 months of age), and the children do not acquire the ability to sit unaided. The main genetic determining factor of the clinical variability of the disease is the copy number of the *SMN2* , an *SMN1* -homologous gene. 3

Effective SMN-dependent therapies, such as gene therapy with *SMN1* gene replacement (onasemnogene abeparvovec) and splicing regulation of exon 7 in *SMN2* (nusinersen, risdiplam), have already been approved by the leading international regulatory agencies and the Brazilian National Surveillance Agency (ANVISA). 4 While nusinersen and risdiplam are approved for use in all clinical forms of SMA-5q, gene therapy is approved for children up to two years of age. Several pivotal and real-world studies have demonstrated the efficacy of these therapies in different clinical forms of SMA-5q. 4 5 6 7 These studies have emphasized the importance of early initiation of therapies, as the earlier treatment starts, the better the outcome. 4 Children diagnosed and treated in the pre-symptomatic phase tend to achieve near-normal motor function. This underscores the urgent need for effective newborn screening programs. 8

In this issue of the *Arquivos de Neuro-Psiquiatria* , Alves et al. (2024), 9 in a paper entitled “Type 1 spinal muscular atrophy cohort before and after disease-modifying therapies” showed that both nusinersen and gene therapy provided substantial benefits in motor, respiratory, and bulbar functions in ten type 1 SMA-5q patients. However, patients with more advanced disease with severe motor and respiratory impairment exhibited less favorable outcomes for any therapies used, as demonstrated in many real-world studies, reaffirming previous findings that highlight the importance of early treatment initiation. 4 Previous studies in Brazilian patients reinforce the efficacy of both gene therapy and nusinersen in treating early-onset and late-onset SMA-5q. 5 6 7

In the study of Alves et al. (2024), 9 among the group treated with gene therapy, there was a more significant functional gain; however, this group started treatment much earlier than the group treated with nusinersen (an average onset of 30.3 months for nusinersen and 15.2 months for gene therapy), confirming that the main determining factor of the outcome is the early initiation of treatment regardless of the copy number of *SMN2* gene. This reinforces the need for early disease diagnosis and rapid access to therapies, especially in the public health system. However, the number of copies of the *SMN2* gene is also a determining outcome factor. Patients with two copies of the *SMN2* gene usually have an earlier onset of disease and a worse outcome than those with three copies of the *SMN2* . In Brazil, the drugs nusinersen and risdiplam are available in the public health system only for patients with SMA-5q types 1 and 2, not for type 3.

The study of Alves et al. (2024) 9 also showed that despite the motor functional gain, respiratory and bulbar functions remained stable during follow-up (90% experienced no respiratory decline, and 30% maintained oral feeding). The stability of bulbar and respiratory functions after treatment, especially in patients with long-term disease, is another well-established data. For example, patients undergoing tracheostomy and gastrostomy tend to remain that way after treatment. 6 However, we must consider that bulbar and respiratory functions are more challenging to assess in children, mainly due to the lack of specific scales and biomarkers to evaluate these functions.

Another critical issue is related to the safety of therapies. In the case of gene therapy, despite the higher risk of complications, these effects are easily manageable, but an experienced medical team must monitor them. 4 Most complications are related to an immune-mediated reaction against the viral vector, which can cause liver disease and thrombocytopenia. Such complications can be prevented in part with the use of steroids in the first months of infusion. It is important to emphasize that the heavier the child, the greater the risk of elevated liver enzymes, causing prolonged steroid use. 7 The nusinersen is an intrathecal medication in which the patient must receive therapy in a hospital setting. The most significant limitation to the use of the treatment is in cases with complex spine (severe scoliosis or previous spinal fusion). Such situations do not preclude intrathecal administration of the therapy; however, it requires special care during its administration, especially with the use of imaging techniques and by experienced personnel. 4

In summary, the findings from Alves et al. (2014) 9 reinforce the efficacy of disease-modifying therapies in SMA-5q type 1, even in advanced cases, while highlighting the critical importance of early diagnosis and timely access to treatment to optimize patient outcomes.
